# Effects of glycyrrhetinic acid β on growth and virulence of *Aeromonas hydrophila*

**DOI:** 10.3389/fmicb.2023.1043838

**Published:** 2023-02-10

**Authors:** Kai Chen, Ting Qin, Liangkun Pan, Xuwen Bing, Bingwen Xi, Jun Xie

**Affiliations:** Key Laboratory of Integrated Rice-Fish Farming Ecology, Ministry of Agriculture and Rural Affairs, Freshwater Fisheries Research Center, Chinese Academy of Fishery Sciences, Wuxi, China

**Keywords:** glycyrrhetinic acid **β**, *Aeromonas hydrophila*, growth curve, virulence phenotypes, *Carassius auratus*

## Abstract

*Aeromonas hydrophila* is a significant pathogen to freshwater farmed animals, and antibiotics are usually used to control the bacterial septicemia caused by *A. hydrophila*. Due to the severe situation of development and spread of antibiotic resistance, there are stricter restrictions on antibiotics used in aquaculture. To evaluate the feasibility of glycyrrhetinic acid β (GA) as an alternative therapy against bacterial infection, in this study, an *A. hydrophila* isolated from diseased fish is used to test the antibacterial, anti-virulence activity and therapeutic effect of GA *in vitro* and *in vivo*, respectively. Results showed that GA did not affect the growth of *A. hydrophila in vitro*, while it could down-regulate (*p* < 0.05) the mRNA expression of the hemolysis-related genes *hly* and *aer*A, and significantly inhibited (*p* < 0.05) hemolytic activity of *A. hydrophila*. In addition, *in vivo* test showed that oral administration of GA was ineffective in controlling acute infections caused by *A. hydrophila*. In conclusion, these findings suggested that GA was a potential anti-virulence candidate against *A. hydrophila*, but the application of GA for the prevention and treatment of *A. hydrophila*-related diseases was still a long way.

## 1. Introduction

*Aeromonas hydrophila* is a widespread opportunistic pathogen in freshwater environment. It is regarded as a significant risk factor in aquaculture because of its high morbidity and mortality (Harikrishnan and Balasundaram, 2007). Antibiotic therapy is still the main measure against *A. hydrophila* infection. However, due to the severe situation in the development and spread of antibiotic resistance, as well as drug residues and environmental pollution (Lulijwa et al., 2019), the alternatives to antibiotics are urgently needed. Anti-virulence strategies are considered as a promising alternative solution (Defoirdt, 2014), which exert less selective pressure on bacteria than antibiotic treatment, and therefore have apparent advantages in reducing the development of resistance.

Glycyrrhetinic acid β (GA), an oleanane-type triterpene, is the main active component of plant *Glycyrrhiza glabra* extract (El-Saber Batiha et al., 2020) and frequently employed in the therapy for human diseases. There are numerous publications on the antimicrobial effects of GA and its derivatives, including antibacterial activity in *Staphylococcus aureus* (Oyama et al., 2016), *Pseudomonas aeruginosa* (Darvishi et al., 2015) and *Helicobacter pylori* (Celik and Duran, 2019). GA is also effective at regulating pathogen virulence, as it can reduce the virulence of *S. aureus* (Li et al., 2012; Long et al., 2013; Huang et al., 2016) and *Streptococcus mutans* (Yamashita et al., 2019), as well as limit the production and release of α-hemolysin by *S. aureus* (Li et al., 2012). Furthermore, GA can effectively reduce pathological damage caused by the pathogen infection (Kim et al., 2013; Long et al., 2013).

Until now, there is limited data about GA in aquaculture, and even the available focus on its impact on farmed animals’ growth, nutrition, and metabolism, as well as non-specific immunity and antioxidation. For instance, *Ietalurus punetaus* (Jiang et al., 2016), *Megalobrama amblycephala* (Cai et al., 2014), *Hypophthalmichthys molitrix* (Harikrishnan et al., 2021), and *Litopenaeus Vannamei* (Chen et al., 2010). Only Schrader (2010) has reported that GA had antibacterial activity against *Flavobacterium columnaria*. To evaluate its potential as a therapeutic agent for prevention and treatment of bacterial diseases, in this study, the antibacterial and anti-virulence activity against pathogenic bacterial *A. hydrophila in vitro* and therapeutic effect *in vitro* of GA were tested.

## 2. Materials and methods

### 2.1. Experimental bacterial strain and fish

*Aeromonas hydrophila* strain employed in this study was isolated from diseased *Carassius auratus*. Gibel carp (*C. auratus gibelio*, 5.3 ± 1.1 g), provided by the experimental station of Freshwater Fisheries Research Center, Chinese Academy of Fishery Sciences, were acclimatized in indoor water recirculation culture system at 28 ± 1°C for 2 weeks. A 2% commercial feed was given twice per day according to body weight. This study was carried out in strict accordance with the recommendations in the Guide for the Care and Use of Laboratory Animals. The protocol was approved by the Committee on the Ethics of Animal Experiments of the Freshwater Fisheries Research Center (Authorization Number: 20220214001). All surgery was performed under MS-222, and all efforts were made to minimize suffering.

### 2.2. Determination of impact of GA on growth of *Aeromonas hydrophila*

Minimal inhibitory concentration (MIC) was determined as previous study (Zhu et al., 2019). GA (97%, Aladdin, China) was dissolved with 100% DMSO to obtain a stock solution of 25.6 mg/mL, then diluted with Nutrient Broth (NB) medium and the final concentrations of GA in culture medium were 256, 128, 64, 32, 16, 8 μg/mL, respectively. *A. hyrophila* at logarithmic growth period was adjusted to 1 × 10^7^ CFU/ml, 50 μL cell suspension was inoculated into 5 mL NB medium with different concentration of GA, negative, solvent and positive controls containing only NB, NB with DMSO and Enrofloxacin (0.1 μg/mL) including bacteria, and then incubated at 28°C for 24 h with shaking (180 rpm). The test was repeated twice, and each group was detected with three parallels. The optical density (OD) of culture at 600 nm was measured using a Multiskan GO spectrophotometer. The MIC was defined as the lowest concentration of the drug that inhibited growth of target bacteria by >90%.

*Aeromonas hydrophila* was inocubated into NB medium with the different final concentration of GA (128 μg/ml, 64 μg/ml, 32 μg/ml, 16 μg/ml, 8 μg/ml, and 4 μg/ml), Enrofloxacin (0.1 μg/ml) and incubated at 28°C. The growth curve was detected with the OD_600_ at 0, 2, 4, 6, 8, 10, 12, 22, 24, and 26 h. The experiment was replicated in triplicate.

### 2.3. Determination of virulence factors of *Aeromonas hydrophila in vitro*

Bacterial cells (1 × 10^7^ CFU/ml, 50 μl) were inoculated into NB medium (5 ml), and cultured with and without GA at 28°C for 24 h with shaking (180 rpm). OD_600_ was determined with a spectrophotometer. The supernatant was collected by centrifugation (10,000 g, 10 min, 4°C), and filtration with a nylon membrane (0.22 μm). The filtrate was stored at −80°C for protease and hemolytic activity assay. The experiment was repeated twice with six parallels in each group.

Azocasein assay was used to detect protease activity as previously reported (Li et al., 2019). Briefly, the filtrate (150 μl) and 0.3% azocasein (1 ml, dissolved in Tris–HCl, pH 7.5) were incubated at 37°C for 30 min, and the reaction was terminated with 500 μl 10% trichloroacetic acid. The supernatant was collected after centrifugation (10,000 g, 10 min, 4°C) and neutralized with NaOH (1 mol/l). Finally, OD_400_ of the supernatant was measured, and the relative activity of protease was calculated as OD_400_/OD_600._

The hemolytic activity was determined as described previously (Li et al., 2019). Briefly, 100 μl filtrate, 100 μl 4% sheep blood cells and 800 μl sterile saline was incubated at 37°C for 30 min. The supernatant was collected by centrifugation (2000 g, 10 min, 4°C) and the OD_405_ was detected. Finally, the hemolysis rate was determined using the formula below: [(sample OD_405_ -blank control OD_405_) / sample OD _600_] / (positive control OD_405_ -blank control OD_405_). By the same way, NB medium with Magnolol (98%, Aladdin, China) at 8 μg/ml was prepared as the positive drug in supplementary tests in hemolytic activity assay (Dong et al., 2017).

The lipase activity was measured using a modified agar-diffusion assay (Zhu et al., 2019). Bacterial cells in logarithmic growth period were adjusted to 1 × 10^5^ CFU/ml and added 50 μl suspension to each well (diameter = 7 mm) in medium (NB base, 1.5% agar, 1% Tween 80) with and without GA and incubated at 28°C. The diameter of the precipitation region was measured at 24 h, 48 h, 72 h, and 96 h, respectively, to indicate the lipase activity. The experiment was done in triplicate, with three parallels in each group.

Motility assay was carried out as in the previous report (Zhu et al., 2019). Bacterial cells in logarithmic growth period were adjusted to 1 × 10^5^ CFU/ml, planted onto the semi-soft medium (NB base, 0.5% agar) with and without GA, and then incubated at 28°C. To determine motility, the colony diameter was measured at 24, 48, 72, and 96 h. The experiment was repeated twice with six parallels in each group.

### 2.4. RNA isolation and gene expression analyzes

For virulence related gene expression analysis, *A. hydrophila* was inoculated in NB medium with and without GA (32 μg/ml), and cultured for 18 h at 28°C with shaking (180 rpm). Each group was conducted with three parallels. Bacterial cells were collected by centrifugation (10,000 g, 10 min, 4°C), washed and diluted with sterilized saline (0.85%), and the equal cell pellets were immediately immersed in RNAiso Plus (TaKaRa) and stored at −80°C.

Total RNA was extracted using a RNAiso Plus kit (Takara). RNA quantities and concentrations were measured using Nanodrop 2000 spectrophotometer (Thermo Scientific), then RNA concentrations were adjusted to 400 ng/μL. cDNA was synthesized using a HiScript® III RT SuperMiX for qPCR kit (Vazyme Biotech, China) following the instructions.

Quantitative real-time PCR was performed on a Bio-Rad CFX real-time PCR detection system (USA) using ChamQ Universal SYBR qPCR Master Mix kit (Vazyme, China). All primers of virulence related genes (Table 1) were synthesized by Sangon Biotech (China), and *recA* was chosen as the reference housekeeping gene (Pang, 2015; Gu et al., 2022). The experiments were conducted in triplicate, and the result was calculated using 2^-△△Ct^ method.

**Table 1:** Primers used for quantitative PCR.

Genes	Primer sequence(5′-3′)	Amplification efficiency
*recA* (Pang, 2015)	CGACCCCATCTATGCCGC	105.7%
	CGACCCCATCTATGCCGC	
*hly* (Gao et al., 2019)	TCTACCTCAACGTCAACCGC	103.0%
	GTCCGCACTATCTTGGCATCC	
*aerA* (Gao et al., 2019)	CACGTCCATGTCTTCACCGA	101.3%
	AGCGCGAATTTCATCAAGCC	

### 2.5. Experimental therapeutics

Gibel carps (180) were randomly distributed into 1 control group and 3 experimental groups (infected only group, infected and treated with GA group, infected and treated with enrofloxacin group). Three parallels were set for each group.

Each fish in experimental groups were injected intraperitoneally with 1% (v/w) *A. hydrophila* suspension (1 × 10^7^ CFU/ml) after anesthetizing with MS-222, while the control group received an injection of sterilized saline. When all fish in tank returned, basic or medicated feed were given to different groups. The control and infected only groups. Were fed with basic feed, while the infected and treated with GA group and the infected and treated with antibiotic group were fed with basic feed supplemented with GA (0.2%, w/w) and enrofloxacin (0.2%, w/w), respectively. Theoretical medication intake was 40 mg/kg of fish (Yang, 2005). The mortality was monitored for 7 days.

### 2.6. Statistical analysis

Statistical analyzes of data were performed in SPSS 23 software. T-test or T’-test was used for data in a normal distribution, while nonparametric test was employed for data not in a normal distribution. The survival curve was analyzed using the Kaplan–Meier method, and the survival rate was analyzed using one-way ANOVA. All data was present as mean ± standard deviation (x ± s), and the significance level was set at 0.05.

## 3. Results

### 3.1. Impact of GA on the growth of *Aeromonas hydrophila*

**Figure 1:**
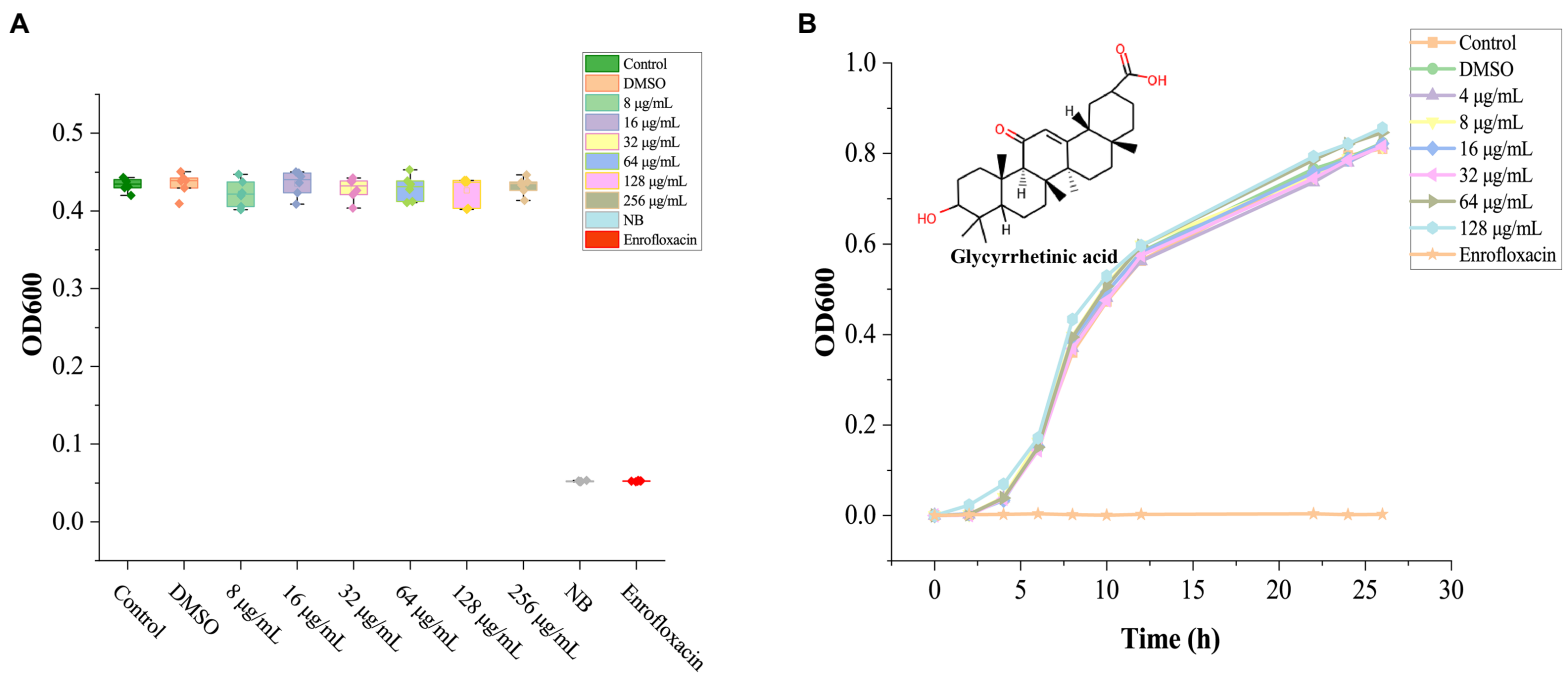
The effect of GA on the growth of *Aeromonas hydrophila.* **(A)** The bioassay results of GA’s MIC against *A. hydrophila* (*n* = 6). **(B)** *Aeromonas hydrophila* growth curves cultured with different doses of GA (*n* = 3). n.s., not significant.

Solvent DMSO (1%) showed no noticeable bacteriostatic effect on *A. hydrophila*, (Figure 1A), GA treated groups with different concentrations all did not show significant inhibition to *A. hydrophila* (*p* > 0.05). Due to the saturated concentration of GA in NB is near 256 μg/ml, therefore the exact MIC of GA to *A. hydrophila* could not be detected here, and, the MIC should be above 128 μg/ml.

The grow curves of *A. hydrophila* in each GA-treated groups were essentially coincident with that in the control group (Figure 1B). The result demonstrated that GA did not affect the growth of *A. hydrophila*.

### 3.2. The effect of GA on the virulence phenotype of *Aeromonas hydrophila*

**Figure 2:**
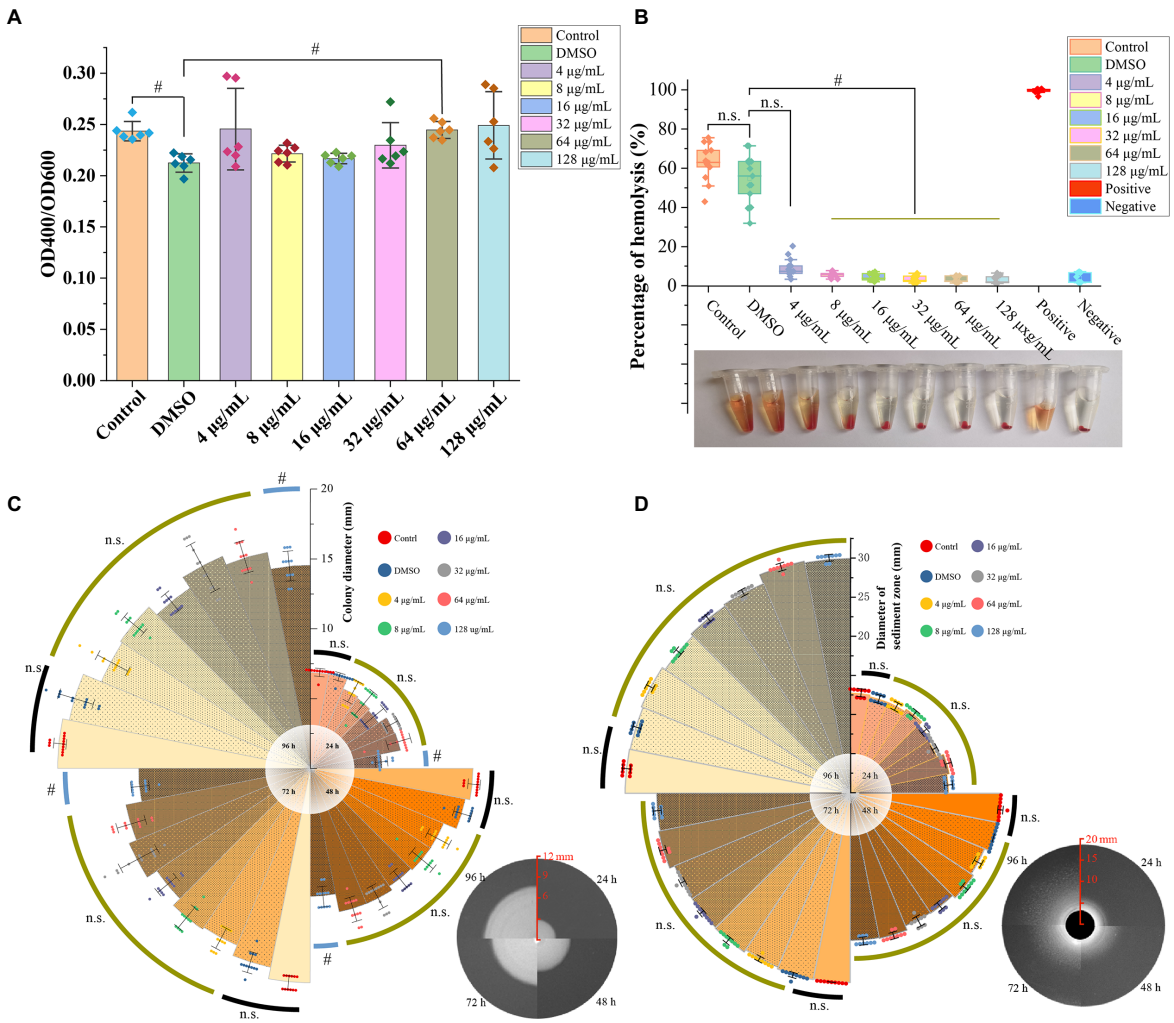
Effect of GA on virulence phenotype of *A. hydrophila.* **(A)** Protease activities of the *A. hydrophila* supernatants co-cultured with different concentrations of GA (*n* = 6). **(B)** Hemolytic activities of the *A. hydrophila* supernatants co-cultured with different concentrations of GA (*n* = 12). **(C)** Motility of the *A. hydrophila* co-cultured with different concentrations of GA (*n* = 12). **(D)** Lipase activities of the *A. hydrophila* co-cultured with different concentrations of GA(*n* = 9). n.s., not significant. #*p* < 0.05.

The azocasein assay revealed that the DMSO significantly decreased the extracellular protease activity compared to the blank control group (*p* < 0.05), however, there was no significant difference between GA treated groups and DMSO group, except one group with GA concentration 64 μg/ml (*p* < 0.05) (Figure 2A). In hemolytic activity, lipase activity and motility tests, DMSO group showed no significant difference compared to the control group (Figures 2B–D) (*p* > 0.05). Furthermore, GA concentration over 4 μg/ml, dramatically reduced hemolytic activity of *A. hydrophila* (Figure 2B). It is generally consistent with the results in the supplementary experiment (Supplementary Figure S1). In aspects of motility, only the highest GA-treated group (128 μg/ml) decreased significantly at 24, 48, 72, and 96 h. According to the result in Figure 2D, there was no significantly difference between all the tested groups at all time points, and GA did not influence lipase activity (*p* > 0.05).

### 3.3. Effects of GA on the expression of hemolysis-related genes in *Aeromonas hydrophila*

**Figure 3:**
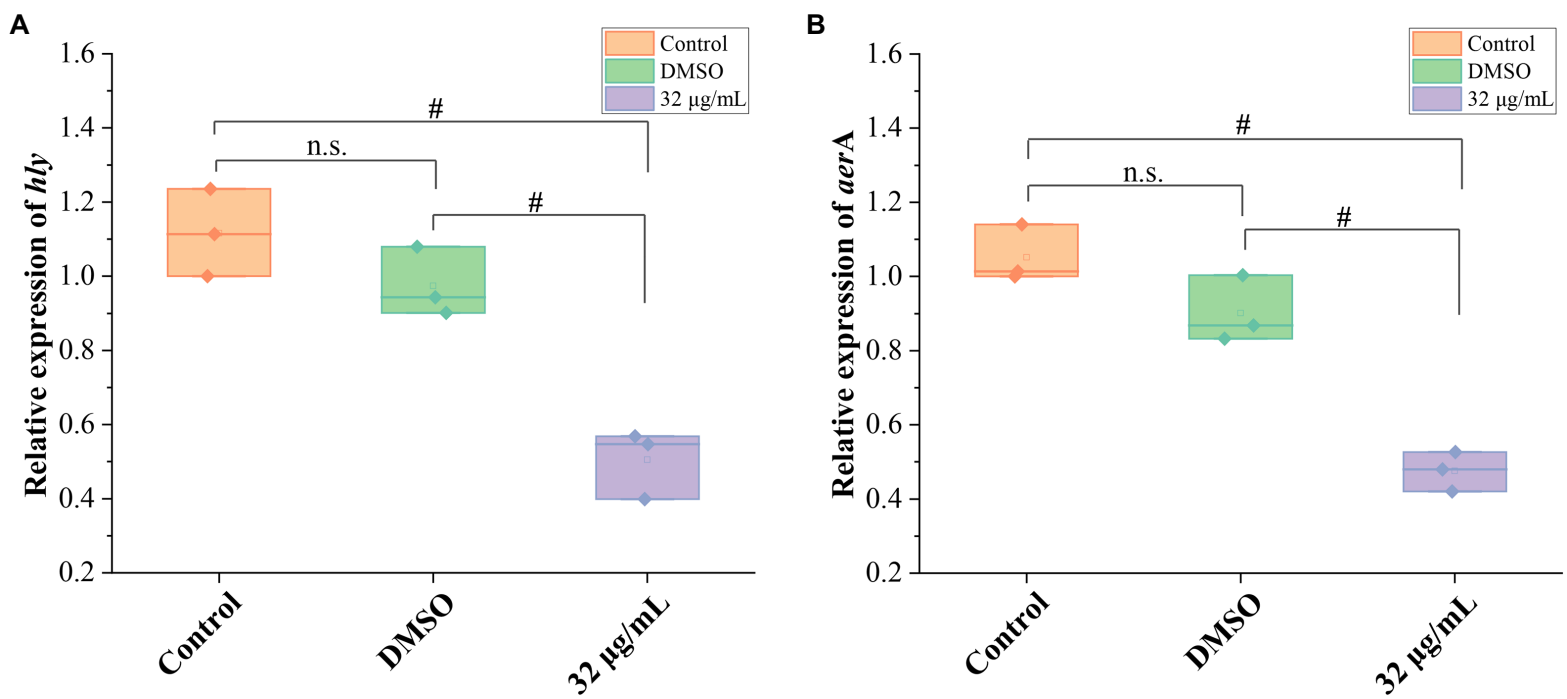
Effects of GA on the mRNA expression of hemolysis-related genes in *A. hydrophila.* **(A)** The relative expression levels of *hly* of *A. hydrophila* GA (*n* = 3). **(B)** The relative expression levels of *areA* of *A. hydrophila* (*n* = 3). n.s., not significant. #*p* < 0.05.

To further confirm the impact of GA on hemolytic activity of *A. hydrophila*, mRNA expressions of hemolysis-related genes (*hly* and *aer*A) were detected. The results showed that the DMSO did not affect the mRNA expressions of *hly* and *aer* A genes, whereas 32 μg/ml GAvsignificantly inhibited the mRNA expression of *hly* and *aer*A genes (Figure 3) and reduced the *hly* and *aer*A mRNA expressions to 51.8 and 52.8% of that in DMSO group, respectively.

### 3.4. Therapeutic effect of GA in goldfish against acute infection of *Aeromonas hydrophila*

**Figure 4:**
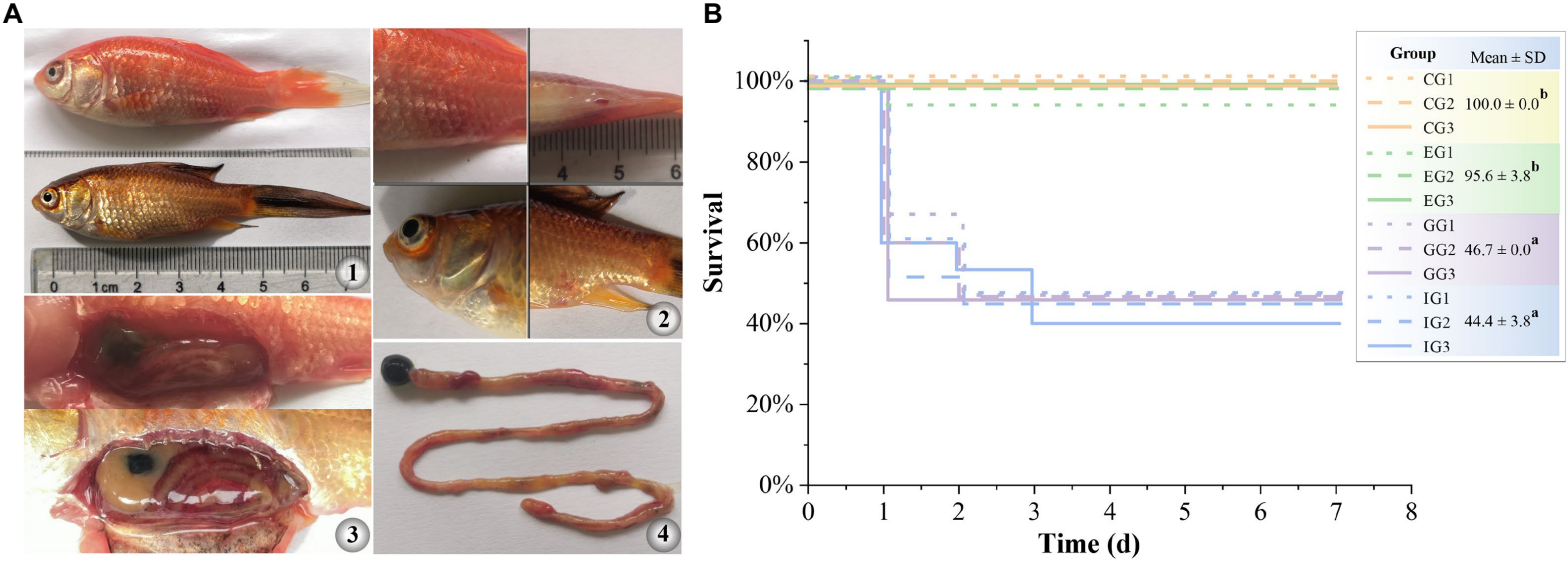
Therapeutic effect of GA in goldfish against acute infection of *A. hydrophila.* **(A)** gross pathological changes (A1, diseased goldfish. A2, symptoms on the body surface. A3, symptoms of internal organs. A4, symptoms of intestinal). **(B)** survival curve (*n* = 15). CG, control group. IG, infected only group. GG, infected and treated with GA group. EG, infected and treated with enrofloxacin group. Different lower-case letters indicated significant differences among groups (*p* < 0.05).

GA significantly reduced the expression of hemolysis-related genes and suppressed the hemolytic activity of *A. hydrophila in vitro*. Therefore, we further tested therapeutic effect of GA in goldfish against *A. hydrophila* infection. The results showed that *A. hydrophila* infection induced acute histopathological damage in goldfish. Moribund goldfish showed redness and swelling around the injection site and cloaca, as well as blooding at the base of fin and at eye (Figure 4A). In addition, autopsy revealed ascites in intestine and body cavity, and enteritis. The survival of goldfish infected with *A. hydrophila* showed no significant difference between the enrofloxacin treated group and that of in control group (*p* > 0.05) (Figure 4B). However, GA treated groups did not markedly increase the survival of goldfish infected with *A. hydrophila* (*p* > 0.05).

## 4. Discussion

### 4.1. Effect of GA on growth of *Aeromonas hydrophila*

In this study, the MIC and growth curve tests revealed that GA had no antibacterial effect on *A. hydrophila*. In *Escherichia coli* (Kim et al., 2002) and *S. aureus* (Oyama et al., 2016), the similar results also have been reported. The effect of GA on the growth of *Bacillus subtilis* was not consistent in different strains (Kim et al., 2002; Wang et al., 2012; Huang et al., 2016). Even it reported that some bacterial strain can use GA as carbon source (Yoshida et al., 2001). GA did not inhibit the growth of *A. hydrophila* isolated from diseased fish, so there was less selection pressure. Therefore, GA employed in aquaculture to control *A. hydrophila* infection will has an advantage in avoiding the development of drug resistance.

### 4.2. Effect of GA on virulence of *Aeromonas hydrophila*

Like many other bacteria, the pathogenicity of *A. hydrophila* is mainly determined by its virulence. Pathogenic *A. hydrophila* usually possess a variety of virulence factors that can mediate the adhesion and invasion of the host tissues, evade host defense mechanisms and contribute to *A. hydrophila* survival and developing an infection. Furthermore, all these virulence factors are frequently associated with their phenotypes, such as hemolytic activity, extracellular enzyme activity and motility. Among these phenotypes, hemolytic activity of *A. hydrophila* is mostly mediated by the expression of *hly* and *areA*. They are the chief exotoxins of *A. hydrophila*, can interfere with host cell physiological activity and injure host tissues and cells directly or indirectly (Sarkar et al., 2021). Extracellular enzymes can degrade host mucus barrier and cause tissues damage, facilitate bacterial invasion into the host tissues (Chen et al., 2019). And the motility of *A. hydrophila* is depended on the structural components, flagella and fimbriae. They play an important role in the first contact between host and pathogen and involve in bacterial colonization and invasion (Qin et al., 2016). The current researches indicate that, inhibiting the expression of protease (Cascón et al., 2000), aerolysin (Li et al., 2011) hemolysis-related genes (Dong et al., 2017), interfering with flagella synthesis (Jiang et al., 2015), or blocking the effect of extracellular enzymes (Wu et al., 2012) and hemolytic exotoxins (Zhang et al., 2015), can effectively reduce the mortality from *A. hydrophila*.

For protection against the development and spread of antibiotic resistance, antibiotics use is becoming more restricted. Therefore, development of virulent-targeted medicines attracts much attention in the prevention and treatment of infection. In this investigation, results showed that GA had little effect on *A*. *hydrophila* lipase, protease, or motility, but did drastically inhibit *A. hydrophila* hemolysis ability, which was similar to these results reported in *S. aureus* (Li et al., 2012; Long et al., 2013; Huang et al., 2016). Furthermore, our results also indicated that GA inhibited *aerA*, and *hly* genes expression, which was consistent with these results in *S. aureus* (Li et al., 2012; Long et al., 2013; Huang et al., 2016). These results suggest that there is a similar mechanism, hemolytic activity is in inhibited *via* down-regulating the expressions of hemolysis-related genes, in these bacteria.

### 4.3. Therapeutic effect of GA on acute infection of *Aeromonas hydrophila* in goldfish

Now, anti-virulence strategies have become an important approach in novel drugs against antibiotic resistant bacterial infections. Then hemolysin and aerolysin as the chief exotoxins are important to the pathogenicity of *A. hydrophila*. Previous researches shown they are promising target for minimizing the virulence of the bacterium. There were some investigated focused on these targets, and have found some medications that had a substantial inhibitory impact on aerolysin and could effectively reduce the mortality of experimental fish from *A. hydrophila*, such as resveratrol (Dong et al., 2021), magnolol (Jiang et al., 2015). We also tested the effect of GA *in vivo*, however, the results were unsatisfactory. Further analysis demonstrated that is probably associated with the pharmacokinetics of different drugs. When compared to Dong (Jiang et al., 2015; Dong et al., 2021) and other researches, GA have little effect on the growth of *A. hydrophila*, the oral administration absorption process may further cause the optimal treatment window missed. Although this experiment was unable to prove GA’s therapeutic effect, we did learn from this failed animal experiment that GA’s application risk in the treatment of acute *A. hydrophila* infection. In the future, we will tweak the program even further and try to assess GA’s therapeutic properties.

## Data availability statement

The original contributions presented in the study are included in the article/Supplementary material, further inquiries can be directed to the corresponding authors.

## Ethics statement

The animal study was reviewed and approved by the Committee on the Ethics of Animal Experiments of the Freshwater Fisheries Research Center.

## Author contributions

BX, JX, and XB contributed to conception and design of the study. TQ and LP participated in sample preparation and processing. KC performed the statistical analysis. KC wrote the first draft of the manuscript. BX, JX, TQ, and LP wrote sections of the manuscript. All authors contributed to manuscript revision, read, and approved the submitted version.

## Funding

China Agriculture Research System of MOF and MARA (CARS-45) National Key R&D Program of China (2020YFD0900300).

## Conflict of interest

The authors declare that the research was conducted in the absence of any commercial or financial relationships that could be construed as a potential conflict of interest.

## Publisher’s note

All claims expressed in this article are solely those of the authors and do not necessarily represent those of their affiliated organizations, or those of the publisher, the editors and the reviewers. Any product that may be evaluated in this article, or claim that may be made by its manufacturer, is not guaranteed or endorsed by the publisher.

## Supplementary material

SUPPLEMENTARY FIGURE S1 Hemolytic activities of the *A. hydrophila* supernatants with different treatments (*n* = 6). n.s., not significant. #, *p* < 0.05.
