# Supplementary figures and images for: Effects of glycyrrhetinic acid β on growth and virulence of Aeromonas hydrophila
Source: Front Microbiol. 2023 Feb 10;14:1043838. doi: 10.3389/fmicb.2023.1043838 (PMC9950564; doi:10.3389/fmicb.2023.1043838)

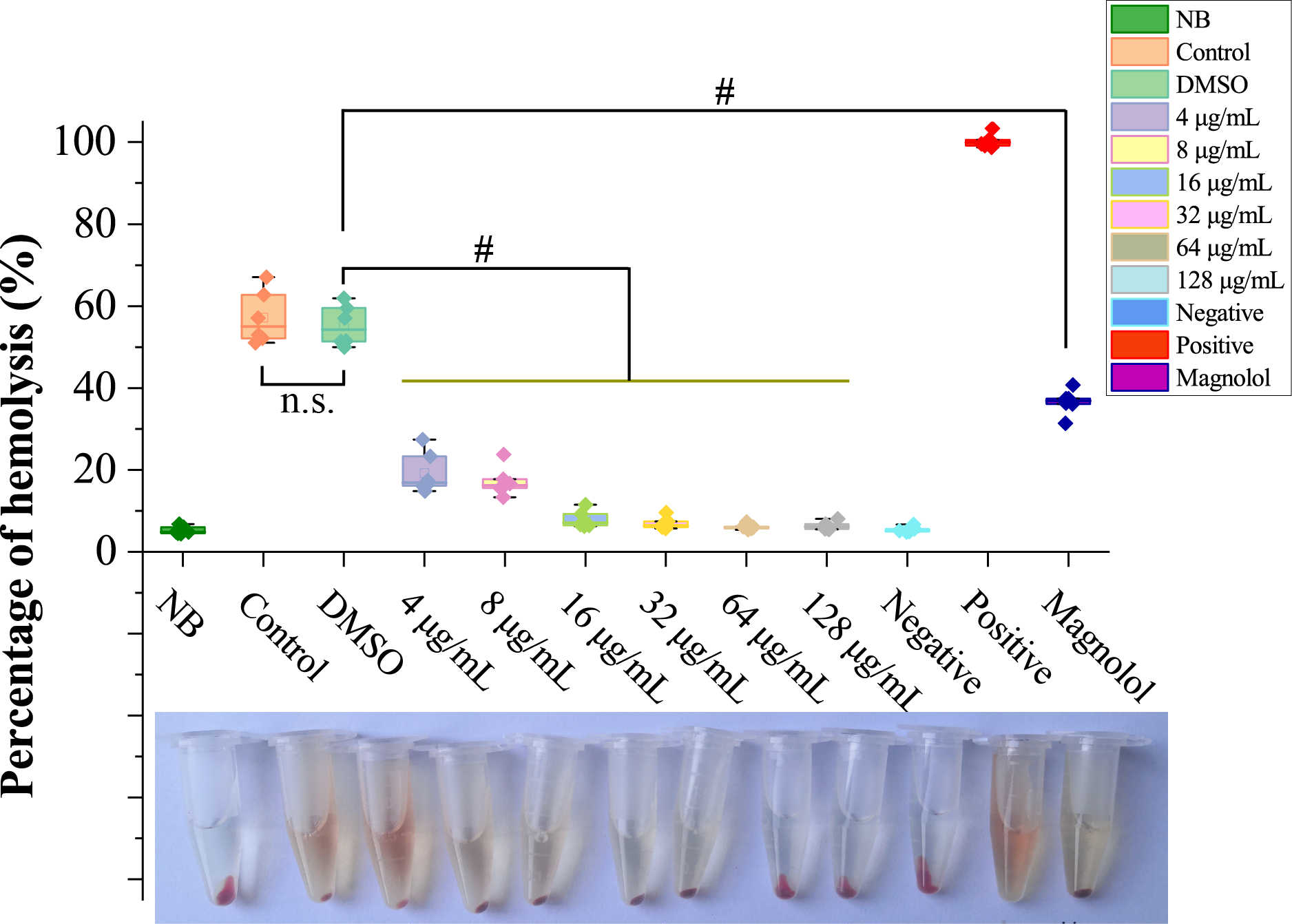

Supplement: SUPPLEMENTARY FIGURE S1 — Hemolytic activities of the A. hydrophila supernatants with different treatments (n = 6). n.s., not significant. #, p < 0.05. [file Image_1.TIF]
